# Green revolution to genome revolution: driving better resilient crops against environmental instability

**DOI:** 10.3389/fgene.2023.1204585

**Published:** 2023-08-31

**Authors:** Rukoo Chawla, Atman Poonia, Kajal Samantara, Sourav Ranjan Mohapatra, S. Balaji Naik, M. N. Ashwath, Ivica G. Djalovic, P. V. Vara Prasad

**Affiliations:** ^1^ Department of Genetics and Plant Breeding, Maharana Pratap University of Agriculture and Technology, Udaipur, Rajasthan, India; ^2^ Department of Genetics and Plant Breeding, Chaudhary Charan Singh Haryana Agricultural University, Bawal, Haryana, India; ^3^ Institute of Technology, University of Tartu, Tartu, Estonia; ^4^ Department of Forest Biology and Tree Improvement, Odisha University of Agriculture and Technology, Bhubaneswar, Odisha, India; ^5^ Institute of Integrative Biology and Systems, University of Laval, Quebec City, QC, Canada; ^6^ Department of Forest Biology and Tree Improvement, Kerala Agricultural University, Thrissur, Kerala, India; ^7^ Institute of Field and Vegetable Crops, National Institute of the Republic of Serbia, Novi Sad, Serbia; ^8^ Department of Agronomy, Kansas State University, Manhattan, KS, United States

**Keywords:** climate change, genome editing, genome revolution, green revolution, marker-assisted selection, omics-assisted breeding, QTL mapping

## Abstract

Crop improvement programmes began with traditional breeding practices since the inception of agriculture. Farmers and plant breeders continue to use these strategies for crop improvement due to their broad application in modifying crop genetic compositions. Nonetheless, conventional breeding has significant downsides in regard to effort and time. Crop productivity seems to be hitting a plateau as a consequence of environmental issues and the scarcity of agricultural land. Therefore, continuous pursuit of advancement in crop improvement is essential. Recent technical innovations have resulted in a revolutionary shift in the pattern of breeding methods, leaning further towards molecular approaches. Among the promising approaches, marker-assisted selection, QTL mapping, omics-assisted breeding, genome-wide association studies and genome editing have lately gained prominence. Several governments have progressively relaxed their restrictions relating to genome editing. The present review highlights the evolutionary and revolutionary approaches that have been utilized for crop improvement in a bid to produce climate-resilient crops observing the consequence of climate change. Additionally, it will contribute to the comprehension of plant breeding succession so far. Investing in advanced sequencing technologies and bioinformatics will deepen our understanding of genetic variations and their functional implications, contributing to breakthroughs in crop improvement and biodiversity conservation.

## 1 Introduction

Agriculture has undergone transformation significantly over the past century as a result of scientific expansion, shifting societal mores and transitions in the political, economic and social environment. Plant breeding is considered to be a co-evolutionary process between human civilization and food sources (Breseghello and Coelho, 2013). From the Green Revolution of the mid-20th century, which aimed to increase crop yields through the use of high-yield varieties to the more recent Genome Revolution, which is leveraging the latest in genomic and biotechnological tools to create crops that are more resilient to environmental instability, the field of plant breeding has been constantly evolving. Over the years, plant breeders and farmers have long been exploiting natural variations to develop and improve crop cultivars (Sadia et al., 2021). However, overexploitation of any practice facilitates disturbances in the balanced ecosystem. For instance, the green revolution in rice in the 1960s led to the invention of the rice cultivar, IR8 (Hargrove and Cabanilla, 1979; Khush, 1999). The widespread adoption of this particular rice variety averted the issue of food shortage across the Globe. However, several socio-ecological concerns were observed with the widespread use of the variety IR8. Several studies have highlighted that IR8 requires a high amount of fertilizer input, which in the long run would cause adverse effects on the environment (Cassman, 1999). In addition, the large-scale utilization of this rice cultivar narrowed down the genetic base, which could result in being more susceptible to certain pests or diseases (Sanchez et al., 2013; Shakiba and Eizenga, 2014). Henceforth, it is undeniable that the excessive crop production generally has a detrimental effect on the environment. But it is also crucial to keep in mind that the scope and character of these effects might be changed based on advanced techniques and technological improvements. It makes it substantially more crucial to carefully consider the benefits and drawbacks of approaches often used in crop development. To address the issues of genetic vulnerability, the application of different advanced molecular breeding tools is highly essential in order to create variability.

**FIGURE 1:**
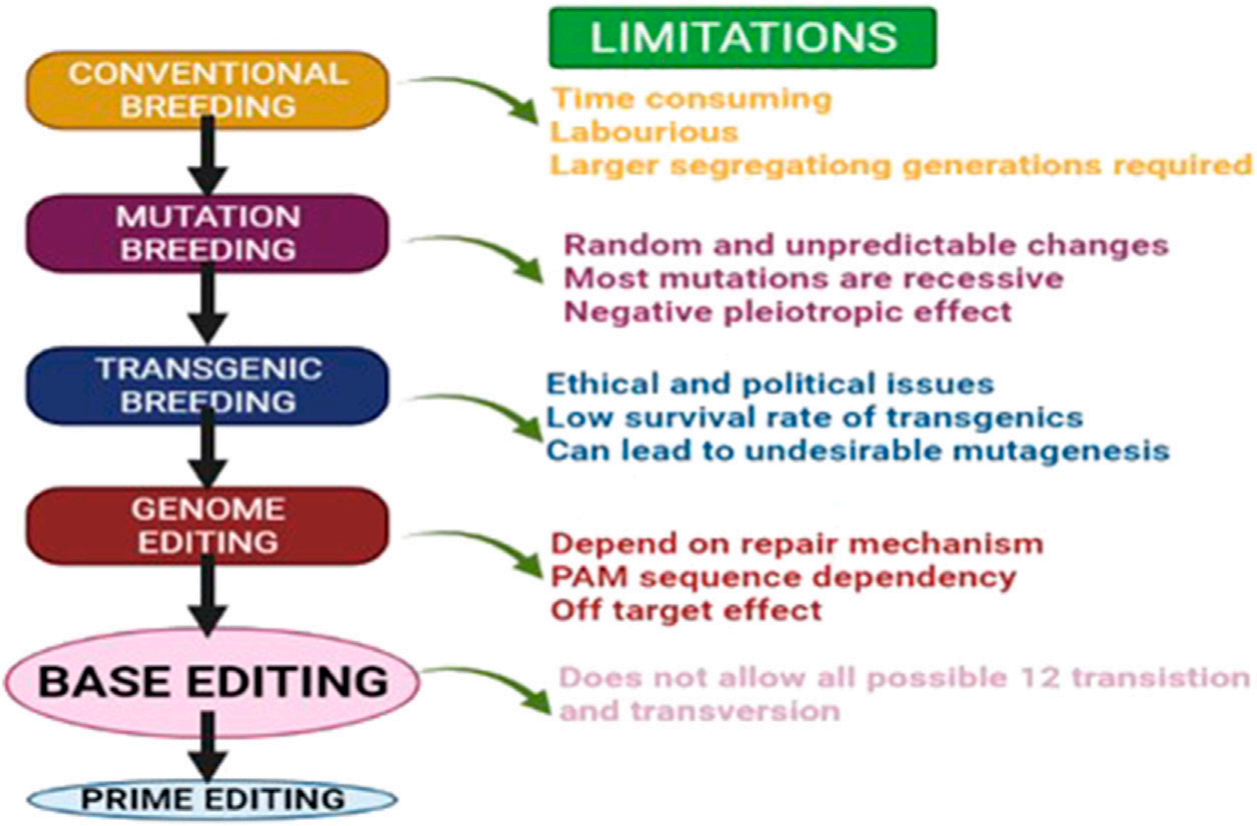
Evolution of major breeding techniques and their limitations.

Among the existing breeding tools, some can create novel variability (Rothan and Causse, 2007; Karthika et al., 2020; Awan et al., 2021; Schleif et al., 2021; Suprasanna et al., 2021) while others can exploit already existing variability (Begna, 2021). The main drawback of conventional breeding is the generation time required for screening potential genotypes over the years in different environmental conditions (Lasley et al., 1994; Prohens, 2011). For instance, mutation as an ultimate source of variation consequently provides better opportunities for novel genetic variability (Holme et al., 2019; Ahmar et al., 2020). However, as a genetic improvement technique, induced mutagenesis has long been an ineffectual approach to obtaining new alleles of genes. This is mostly due to the necessity to generate and evaluate massive populations of presumed mutants, the prevalence of chimaeras, and the recessive character of mutations (Mba, 2013). With the advancement of biotechnology, via the transgenic breeding approach, successful desirable gene introduction into the host organism has now been possible (Visarada et al., 2009). However, the environmental and ethical issues have also imposed problems related to the release of transgenic varieties (Dale et al., 2002; Snow et al., 2005). Numerous reports from different committees have provided clear evidence that contradicts the notion that transgenic crops had a detrimental impact on the environment. (Genetically Engineered Crops: Experiences and Prospects, 2016). But again, disagreements among experts have imposed issues with the global dissemination of transgenic crops. To create desirable variability in our crops, a more sophisticated and time saving technology is indeed required (Figure 1). The rise of molecular approaches such as marker-assisted breeding (MAB), genome wide association mapping (GWAS), targeting induced local lesions (TILLING), and others has rendered it simpler to evaluate potential genotypes. Breeding schemes such as speed breeding (SB) and rapid generation advancement (RGA) were developed to further expedite generation advancement.

Along with the technological progression, the DNA/RNA repair mechanisms viz., homologous direct repair (HDR) and non-homologous end joining (NHEJ), genome editing techniques were uncovered to create double-strand breaks. Over the years, genome editing techniques have evolved, from mega nucleases (MegNs) to zinc finger nucleases (ZFNs) and transcription activator-like effector nucleases (TALENs). Recently, the genome editing technique; clustered regularly interspaced short palindromic repeats (CRISPR) made a scientific breakthrough for agricultural applications. ZFNs and TALENs were regarded as first-generation (Razzaq et al., 2019) while CRISPR is considered as a second-generation editing tool and further revolutionized precision for genome editing (Jinek et al., 2012; Doudna and Charpentier, 2014). For instance, speed breeding-CRISPR is one of the techniques that can be implemented to hasten the generation cycle and development of new cultivars (Samantara et al., 2022). The primary objective of this review is to discuss the overall aspects starting from the conventional breeding approaches to the modern breeding tools (classified in different phases) considering the ease of use and hastening the entire breeding programme in order to generate novel desirable crop cultivars. This review also highlighted previously applied tools, their drawbacks, mitigating strategies and evolving technologies in the field of plant breeding. Furthermore, the discussion here aims to investigate how plant breeders are utilizing the most recent scientific tools and methods to cultivate crops that are better equipped to survive the difficulties encountered by an evolving environment and a growing population.

## 2 Phase I: unleashing nature’s potential: conventional plant breeding

Plant breeding is critical in addressing our population’s rising food demand along with upgrading nutrition (Borlaug, 1983; Huang et al., 2002; Bouis and Saltzman, 2017). In addition, it has given us the ability to develop and improve existing cultivars that are better adapted to climate change and new disease strains. It has been established that the domestication of food and animal crops progresses together with human civilization (Gupta, 2004). Historically, plant breeding has evolved from conventional to genomics-assisted breeding. Conventional breeding entails selection through phenotypic evaluation. Breeders use crossing strategies to combine favorable attributes (reside in the genetic background) from various yet related plants to a new variety (Acquaah, 2015). The screening of certain traits would largely be from a large set of populations. However, this type of breeding process is laborious and time-consuming.

**FIGURE 2:**
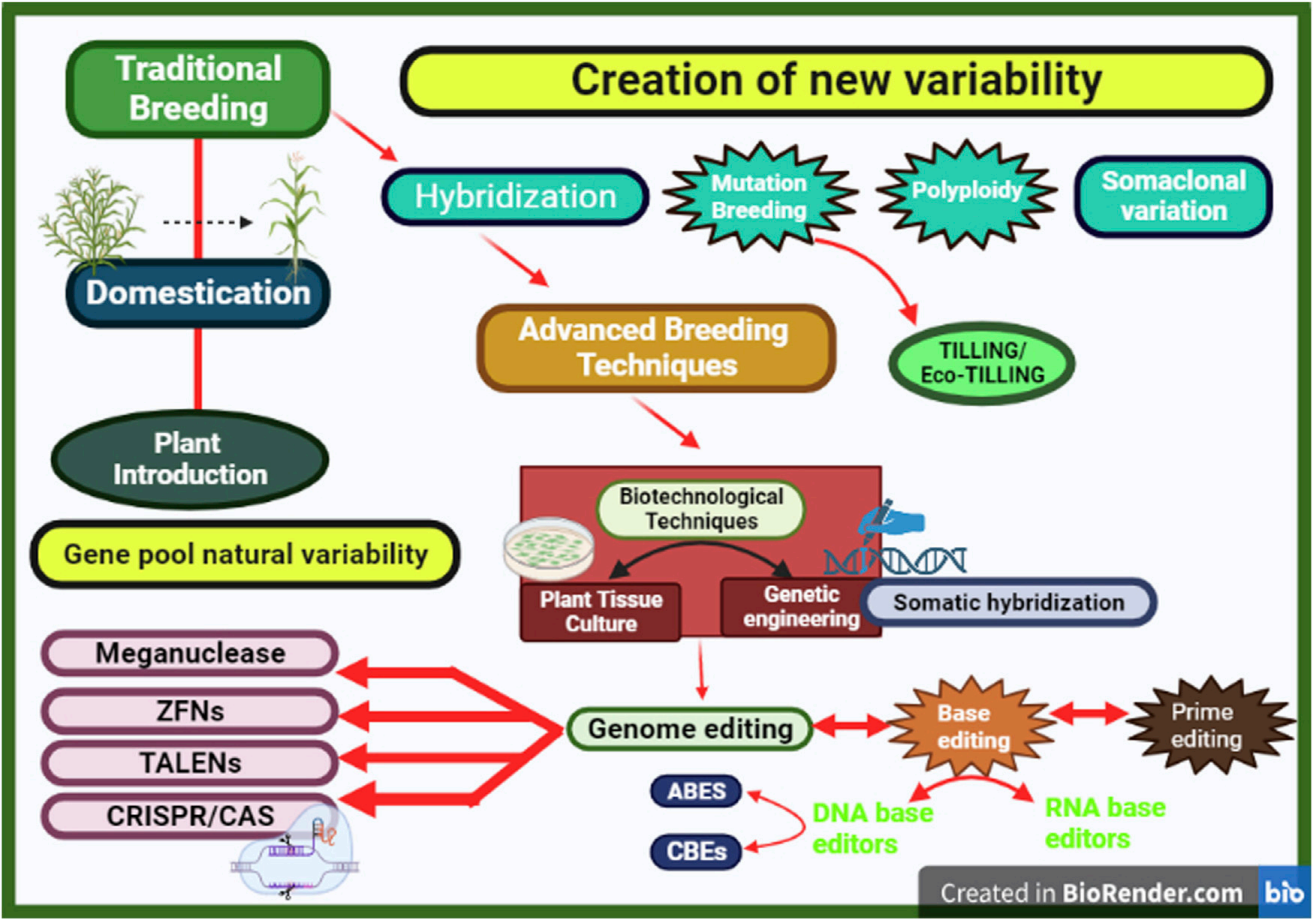
Breeding techniques across eras: tracing advances from tradition to innovation.

Mutation breeding contributed significantly to crop breeding by creating evolutionary divergence (Yu et al., 1991; Kharkwal et al., 2004; Oladosu et al., 2016). This has been successfully used across various crops such as maize, barley, rice, lettuce, and other crops (Stadler, 1928a; 1928b; Sawada et al., 2016; Yamatani et al., 2018). As compared with conventional hybridization, mutation breeding has a shorter time frame for varietal development (Saima Mir et al., 2021). Breakthroughs in breeding have led to the discovery of novel technologies for eliciting variation in target crops. Mutation breeding is still utilized in conventional modern breeding today, despite the more sophisticated procedures given by contemporary biotechnology (Bado et al., 2013). Succeeding this method are other advanced techniques such as Eco-TILLING, plant tissue culture, and genetic engineering (Figure 2).

### 2.1 Crop domestication

**FIGURE 3:**
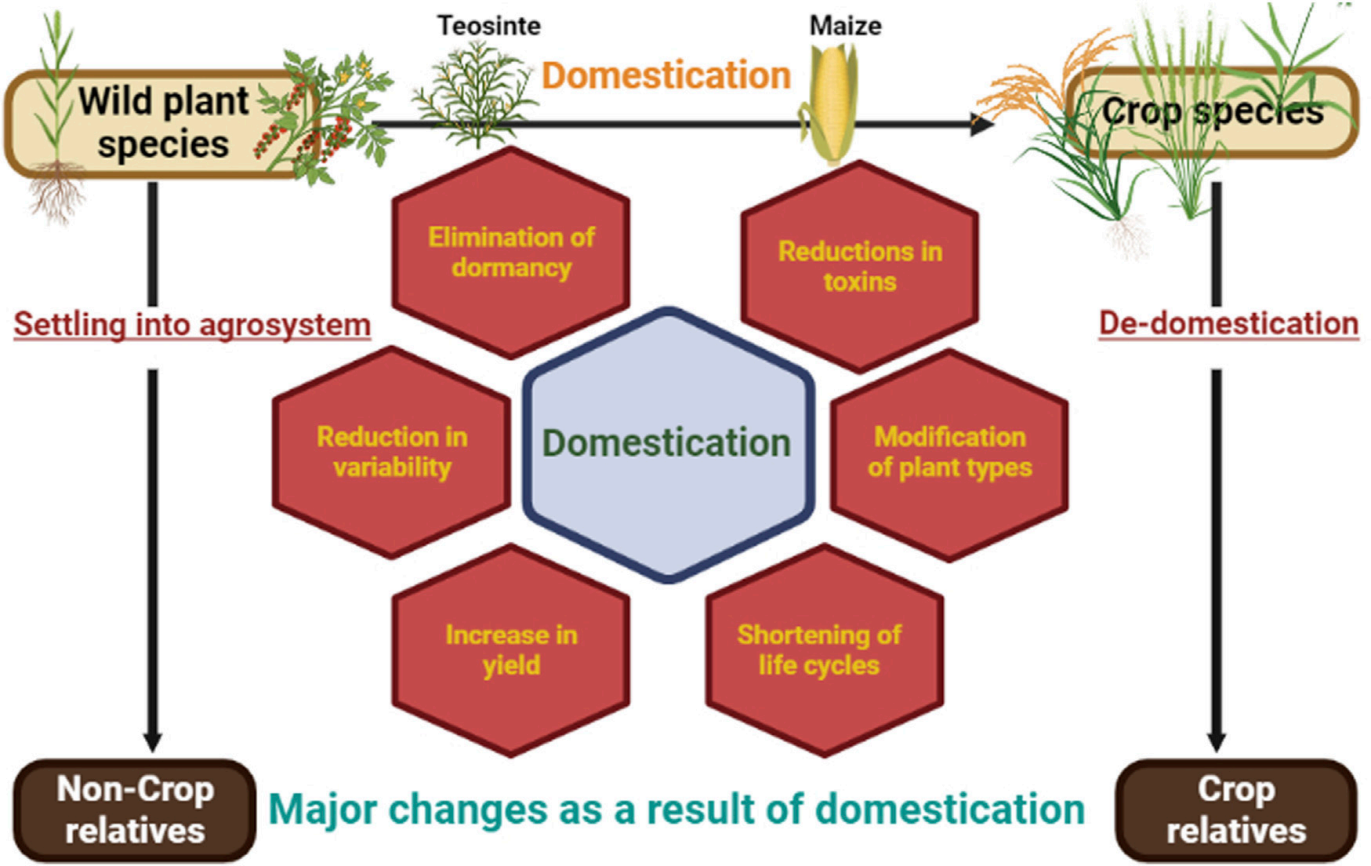
Domestication and de-domestication: transformations in plant traits.

Domestication is a process of coevolution where wild species were brought under human cultivation through artificial selection which led to the differentiation and development of new species (Fuller et al., 2014; Schaal, 2019; Chen et. al., 2021a). The transformation occurred many times independently around the world during the Neolithic period (Childe, 1949), where hunter-gatherer tribes were settled as agricultural societies (Diamond, 2002; Diamond and Bellwood, 2003) and manipulated the rise of modern civilization (Chen et. al., 2021a). The spontaneous mutation had a significant role in modifying the attributes of domesticated plants. Significant changes in necessities, such as land for agriculture, food demand, and the use of agricultural technologies, came from the coevolution of civilization and the human race. As a result, the necessary crops were eventually expanded from their restricted growing zone to be produced in a newer, undiscovered location. As a result, wild plant species were cultivated and favored plants were chosen based on their requirements. Although many initiatives are underway to broaden the genetic background of prominent crops by introgression of genes from their wild relatives (Sadiki et al., 2007). Yet there is a considerable gap slot in the knowledge of forbears, origin and domestication time frames for different crops (Meyer et al., 2012). To get a fuller insight into these unanswered concerns regarding domestication, genomics and related disciplines (transcriptomics and epigenomics) have resulted in new findings (Barrera-Redondo et al., 2020). This demonstrates a bridge between traditional and modern breeding approaches. One of the aspects of domestication is de-domestication, also known as feralization, which is a fascinating phenomenon in which there is a deliberate establishment of a population of domesticated crops in the wild (Figure 3). De-domestication research has helped in better grasping the complexities of crop evolution, as well as the genetic novelties of de-domesticates that are useful in current crop breeding (Wu et al., 2021).

Humans have domesticated different plants in need of food, fodder, fibre and tools throughout the past 12,000 years, which has had an influence on human culture as well as germplasm under domesticated species. Domestication is significant since it makes plant species more accessible to agricultural development programmes. During the phase of domestication, there is the role of artificial as well as natural forces in selection which leads to major changes (domestication syndrome) (Hammer, 1984) (Figure 3). This includes for instance, 1) loss of dormancy [in Chenopodium, the outer layer of the testa is accountable for the black appearance of wild seeds which is diminished or nonexistent in domesticates, resulting in pale-colored seeds (Wilson, 1981; Bruno, 2006)] 2) reduced toxins (wild potato tubers contain amounts of bitter glycoalkaloids that may be hazardous to humans (Johns and Alonso, 1990)] 3) increase in size (Pericarps and placentas of near-isogenic tomato fruits with small vs. big fruits have cells of similar size, but the giant fruits have more cells (Cong et al., 2002)]. Therefore, domestication is regarded as the most crucial and centric method of plant breeding (Table 1) since all other breeding methods become relevant only after it has been domesticated with success. Many of the crops under current cultivation have been domesticated from ancient times. It alleviates a significant number of problems posed by intensive agriculture. A better comprehension of the development of adaptation among crop species might lead to novel ideas for developing new varieties/species that can address present and potential environmental challenges sustainably (Purugganan, 2019).

**TABLE 1:** Application of different classical techniques in crop improvement.

Crops	Traits improved	Target genes	Remarks	References
Domestication				
Rice	Plant architecture	Prostrate growth 1 (*PROG1*)	Upright plant growth	Jin et al. (2008)
				Tan et al. (2008)
	Number of grains per panicle	Frizzy panicle (*FZP* gene	Boosted number of secondary branches	Huang et al. (2018)
	Seed shattering	*SHA1* (a single dominant gene Shattering 1)/*SH4* (Shattering 4) and QTL present in chromosome 1(*qSH1*) controls seed shattering	Reduced shattering of seeds	Li et al. (2006)
				Konishi et al. (2006)
				Lin et al. (2007)
				Sweeney and McCouch (2007)
				Vaughan et al. (2008)
	Seed dormancy	Seed dormancy 4 on chromosome 7 and *OsG* on chromosome 4	Genes governing seed dormancy	Sugimoto et al. (2010)
				Wang et al. (2018a)
Maize	Apical dominance	*tb1*gene (Teosinte branched1)	Regulating the inflorescence architecture	Doebley et al. (1997)
				Cubas et al. (1999)
	Increase in ear	*zfl2* (Zeafloricaula leafy2)	Increase in ear rank number	Bomblies and Doebley (2006)
	Sexual conversion	*tru1* gene (tassels replace upper ears1)	Conversion of a tassel in teosinte to an ear	Doebley et al. (1995)
				Dong et al. (2017)
				Chen et al. (2019)
Selection and hybridization				
Maize	Inflorescence	Remosa 1 (*ra1*) gene	Ear and tassel are suppressed	Vollbrecht et al. (2005)
				Sigmon and Vollbrecht (2010)
				Xu et al. (2017)
				Chen et al. (2019)
Hybrid crop breeding/cross breeding				
Rice	Yield increases		Cross Breeding	Witcombe et al. (2013)
	Increased spikelet number per panicle			Panigrahi et al. (2019)
Wheat	Grain yield			Basnet et al. (2019)
Mutation breeding				
Wheat	Dwarf height of plant	*Rht-B1b* & *Rht-D1b*	Single base pair mutation	Peng et al. (1999)
				Ellis et al. (2002)
	Rust resistance	*Lr34*	SNPs	Dakouri et al. (2010)
				Krattinger et al. (2009)
				Chauhan et al. (2015)
Rice	Increased 1000-grain weight	*GW2* gene	Loss of function mutation	Song et al. (2007)
	Higher nitrogen use efficiency	*NRT*	*NRT1*.*1B* (SNP)	Hu et al. (2015)
				Li et al. (2018a)
	Disease resistance (bacterial blight)	*Os8N3*	Loss of function mutation	Kim et al. (2019)

### 2.2 Crop introduction

The plant has been introduced when it has been brought by humans across a vast geographic border (Richardson et al., 2000). Richardson et al. (2000) differentiated between introduction and invasion, proposing invasion as being spontaneous and without human intervention [earlier supported by ecologists (Clement and Foster, 1994)]. It is one of the easiest methods to use naturally existing variability from one area to be introduced in an area of absence. Plant variety to be introduced in new areas can be used directly as an elite commercial variety without any alteration in its genotype, referred as primary introduction. On the contrary, for secondary introduction, the cultivar is reinforced in selection and hybridization programmes and modified with some new characters. A classic example of introduction is the dwarf wheat variety Sonora-64 and Lerma Rojo as a primary introduction and Kalyan Sona and Sonalika as a secondary introduction (Allard, 1999). Although it is a beneficial method in adding variation to the gene pool in particular areas, some instances in history which led to the introduction of pests, weeds and fungal diseases along with crops unintentionally (Knezevic, 2017)**.**

### 2.3 Crop selection

The differential rate of reproduction of different genotypes is referred to as selection (Lennox and Wilson, 1994). This can be performed in the presence of variability. However, the effectiveness of selection is mainly dependent on the presence of variability since selection cannot create new variations and heritability of the trait (Gregory, 2009). Evidence for selection can be seen from ancient times when farmers used this in agriculture. Selection in crop varieties has been reported by agriculturists, viz., Vans Mons in Belgium, Andrew Knight in England and Cooper in the United States. Publications from 1843 show the evidence for selection practiced in the Isle of Jersey. Individual plant selection was practiced by Patrick Shireff in wheat and oats at the same time (Sneep, 1966). Hallet also reported individual plant selection in wheat, oats and barley in the year 1857. Vilmorin came up with the individual plant selection with progeny testing in the later years (Vilmorin, 1930). Artificial selection represents a sophisticated way of establishing the underlying genetic variation and hence the evolvability of certain features (Table 1). It imparts a known intensity and direction of selection to specific morphological characters (Gregory, 2009).

### 2.4 Crop hybridisation

The genetic base of our current crop population has been narrowed down, hence the need to create variation. The process of hybridization aims to create variation in the population (Anderson and Stebbins, 1954). In layman’s terms, this refers to a method of crossing between individuals of different plant populations that differ from each other in their heritable characters (Harrison, 1990). The process involves crossing two distinct genotypes, resulting in a Filial 1 (F_1_) hybrid. Within the F_1_ generation, genes segregate and recombine, leading to the formation of new gene combinations and thereby generating genetic variability. Different methods can be used; however, most continued inbreeding is done in the lines taken from the source population which is coupled with selection. Hybridization allows exploiting of heterosis (Timberlake, 2013). However, several factors may affect hybridization frequency such as reproductive barriers and incompatibilities between populations and taxon-specific differences.

### 2.5 Mutation breeding

**FIGURE 4:**
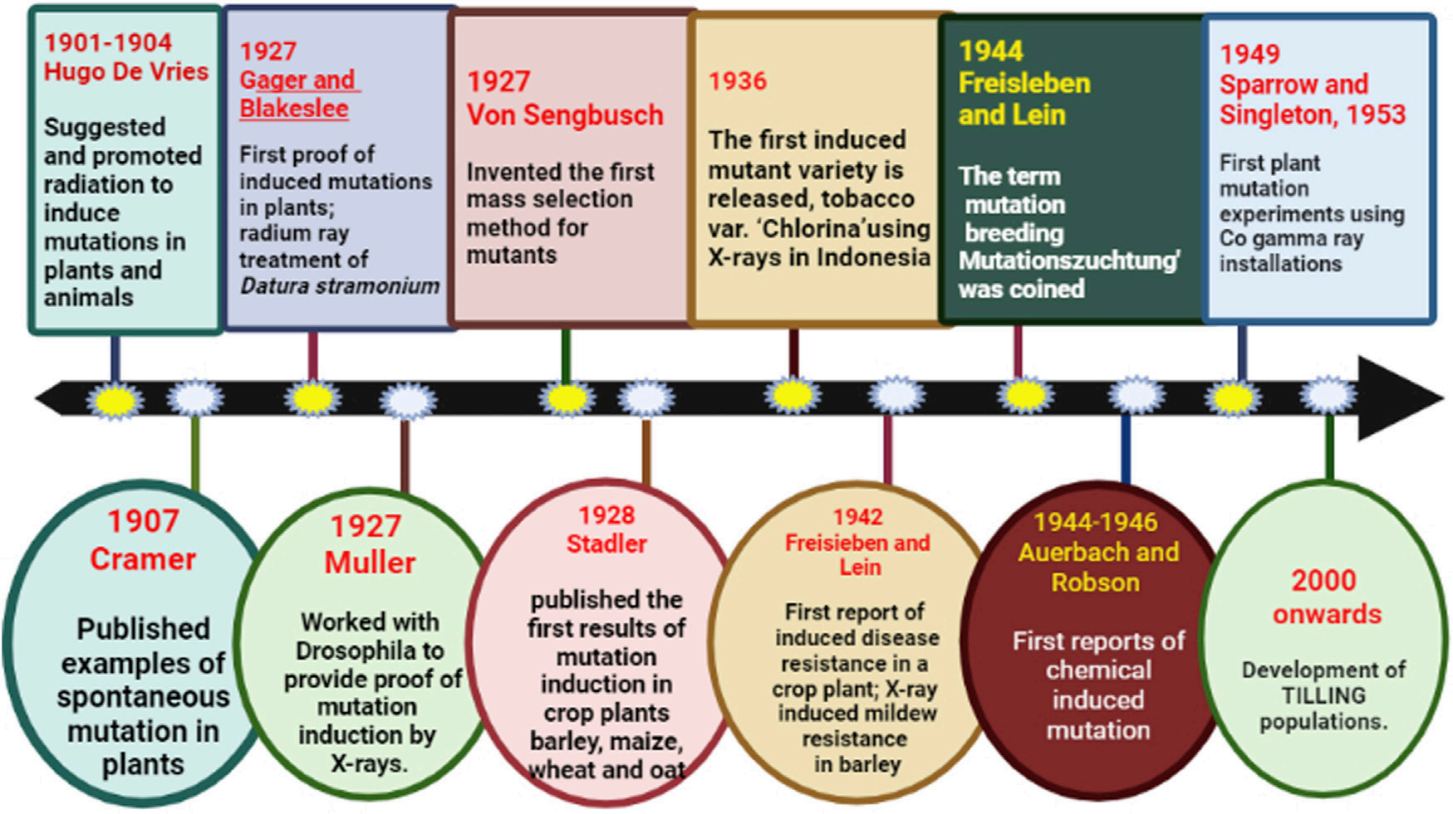
Timeline of milestones in mutation breeding.

Sudden heritable change in the characteristics of the organism that has not been acquired by genetic recombination is referred to as mutation (Van Harten, 1998). The term mutation breeding (“Mutationszuchtung”) was first coined by Freisleben and Lein (Figure 4) in the year 1944 which refers to the development of mutant lines to improve crops (Freisleben and Lein, 1944). Crop improvement can only be achieved when enough variation is present for the trait to help the breeder to exercise his breeding skills. However, because of the overexploitation of commercial varieties, the genetic base has narrowed down. Even if the desired variation is present, it is present in wild relatives/old landraces which makes breeding tedious and time taking to retrieve this variation. Mutation breeding provides an opportunity without extensive upgrading crossing and selection (Shu et al., 2012; Rani et al., 2016). Induced mutagenesis serves as one of the most productive strategies for creating evolutionary divergence as well as identifying critical regulatory genes for commercially significant features for crop improvement (Mehta and Basha, 2018; Chaudhary et al., 2019a). Forward genetics as well as reverse genetics both played crucial roles in achieving significant improvement for various economic traits (Aklilu, 2021). The former involves the identification of an induced or random mutant gene that is responsible for a particular phenotype, while in the latter the function of a gene is not known (Jankowicz-Cieslak and Till, 2015).

Gene inactivation has been implemented extensively to figure out the role of the unknown gene in different crops. To inactivate endogenous genes, T-DNA or transposon is injected into the gene to modify and tag it (Munoz-Lopez and Garcia-Perez, 2010; Kyndt et al., 2015). Insertional mutagenesis in crops is commonly used in crop improvement, particularly for creating disease-resistant varieties. The inserted DNA often contains genes that confer resistance to specific pathogens, making the plant more resistant to disease (Gachomo et al., 2003; Webb et al., 2006). However, insertional mutagenesis can also have unintended consequences, such as altering the expression of important genes and leading to unexpected phenotypic changes in the plant (Miki et al., 2009; Schnell et al., 2015). In addition, these techniques require effective plant transformation, which is only accessible in a few crops, prominently maize and rice (Kolesnik et al., 2004). This led to the discovery of a new approach, “TILLING,” which is now preferred over other methods of reverse genetics.

Although physical and chemical-induced mutagenesis have expedited crop improvement (Mba et al., 2010; Mostafa, 2011; Kozgar et al., 2012), these approaches are still less popular due to random mutation and its expensive nature. In contrast, innovations in genome editing techniques have reimagined the ability to accurately manufacture a specific modification in the genome.

## 3 Phase II: molecular and advanced plant breeding technology

### 3.1 Marker-assisted selection

In today’s world, molecular markers provide significant potential in terms of systems, creative and enhanced genetic mapping methodologies. In 1986, the first authentic restriction fragment length polymorphism (RFLP) map in an agricultural crop (tomato) was generated (Bernatzky and Tanksley, 1986). The advantages of MAS included assessing plants at the beginning of the growing season, screening several qualities that would ordinarily be epistatic with one another, deterministically removing linkage drag, and fast retrieving the genotype of a recurring parent (Tanksley et al., 1989). Even the technological constraints of early RFLPs seemed to be overcome as newer and simpler molecular marker systems like RAPDs (Williams et al., 1990) AFLPs (Vos et al., 1995), and microsatellites (Akkaya et al., 1992) were developed. Within a few years, high-density DNA marker maps for nearly every key crop species had been created (O’Brien, 1993), each one promising the use of strategic MAS to supplement, if not replace, traditional plant breeding strategies. This allows plant breeders to make more informed decisions about which plants to use in their breeding programs, leading to faster and more effective improvement of crops (Garrett et al., 2017; Boopathi, 2020). MAS can also be used to reduce the threat of inadvertently integrating undesirable characteristics into the genome (Bhatnagar-Mathur et al., 2015). Soon after, QTL mapping rose to prominence, facilitating the precise mapping of genetic loci influencing complex characteristics (Phillips and Vasil, 2003). Before the emergence of molecular markers, the concept of promptly identifying the loci driving polygenic characteristics appeared to be a utopian dream.

### 3.2 QTL mapping

Quantitative trait locus (QTL) mapping is a strong technique for the investigation of complex attributes in plants. It is the way of discovering particular locations in the genome that are accountable for variation in a certain characteristic of relevance (Mather, 1938). QTL mapping involves several steps, including the identification of phenotypic variation in a population of individuals, the genotyping of those individuals, and the statistical analysis of the genotype-phenotype data to identify QTLs (Dhingani et al., 2015). The first step in QTL mapping is to generate a mapping population, which can be done by crossing two parental lines with contrasting phenotypes or by selecting individuals from a natural population with phenotypic variation.

Once the mapping population is generated, the phenotypic data is collected, and the individuals are genotyped using molecular markers, such as SNPs or SSRs (Park et al., 2021). The markers are used to construct a genetic map of the genome, which provides a framework for the location of the QTLs. The next step is to perform a statistical analysis of the genotype-phenotype data to identify QTLs. The most commonly used method for QTL mapping is linkage analysis, which compares the phenotypic data with the molecular markers to identify regions of the genome that are associated with the phenotypic variation (Akond et al., 2019). Once the QTLs are identified, further fine-mapping can be done to narrow down the chromosomal region relevant to the trait variation (Raihan et al., 2016b). QTL mapping has been used effectively in various crops, including rice, maize, and tomato, and has revealed insightful information into the genetic basis of complex characteristics including yield, biotic and abiotic stress resistance (Bai et al., 2018; Yadav et al., 2019; Saleem et al., 2020; Goering et al., 2021). In addition, QTL mapping has also been used to identify candidate genes for the targeted improvement of crops (Luo et al., 2019b; Guo et al., 2019; Kumar et al., 2020). In conclusion, QTL mapping provides valuable information for plant breeding and improvement, as well as for understanding the genetic basis of phenotypic variation.

The acronym MAGIC stands for “Multiparent Advanced Generation Inter-Cross,” and it refers to the process of establishing a population by crossing many distinct founder parents across several generations in order to harness the genetic diversity contained in the founding lines (Samantara et al., 2021). Magic populations provide higher genetic variety and improved possibilities for identifying the genetic basis of complex characteristics by mixing many founding parents and allowing for recombination over several generations (Huang et al., 2015). Magic populations are exceedingly helpful for quantitative trait mapping and genetic research. The increasing genetic diversity and the enormous number of recombination events in these populations improve the ability to discover and map quantitative trait loci (QTL) associated with complex characteristics (Huynh et al., 2018; Diaz et al., 2020).

### 3.3 Association mapping (AM)

The idea of linkage disequilibrium (LD) underpins association mapping, which is the non-random association of alleles (gene versions) at distinct loci (positions) on a chromosome (Joiret et al., 2019). In the context of association mapping, LD refers to the fact that some genetic variations tend to occur together more frequently than expected by chance. Association mapping plays a crucial role in crop improvement by identifying the genetic variation underlying traits of interest in crops (Zhu et al., 2008). For example, association mapping studies have identified genes that regulate grain size and weight, which are important components of yield in cereal crops (Cockram et al., 2010; Neumann et al., 2011; Sukumaran et al., 2012; Alipour and Darvishzadeh, 2019; Alqudah et al., 2020) The goal of association mapping is to identify the specific genetic variations that are most likely to be associated with a particular phenotype, such as height, weight, or susceptibility to a disease or abiotic stress (Poland et al., 2011; Sehgal et al., 2015; Hanson et al., 2018). The approach is further expanded to minor millets, offering a food security perspective (Babu et al., 2014; Gupta et al., 2014; Puranik et al., 2020). The method can be used to identify novel alleles and genes that can be used to improve crops through conventional breeding methods or genetic engineering.

Additionally, association mapping enables researchers to identify the relationships between the genetic makeup of crops and the environment, allowing for the development of more sustainable and resilient crop varieties. This information can be used to design breeding programs that are better suited to the specific environmental conditions faced by farmers. Some of the main limitations of association mapping (Korte and Farlow, 2013; Gupta et al., 2019a) include false positive results (AM can sometimes produce false positive results, which means the association between a particular genetic variation and a trait may be coincidental, rather than causal, also may be due to confounding factors, such as population structure, or to multiple testing errors), low statistical power (AM studies often have low statistical power, that they are not always able to detect the true associations between genetic variations and traits, Missing data (AM requires high-quality genotyping data, which is not always available and missing data can result in reduced statistical power or can lead to the exclusion of important individuals from the analysis), Complex trait analysis (these are often influenced by multiple genetic variations, some of which may be rare or have small effects). AM is not well suited for analyzing these traits, as it can only identify common genetic variations that have moderate to large effects, Cost and time: large numbers of individuals are required to be genotyped and phenotype.

Integrating association mapping with a secondary mapping population is a powerful strategy to validate and refine the findings of association mapping. Secondary mapping populations have been essential in enhancing crop breeding efforts in a variety of crops. Secondary mapping populations, for example, have proved useful in mapping and dissecting complex characteristics. Researchers found quantitative trait loci (QTLs) linked with these qualities by crossing divergent parental lines and analyzing the resultant segregating populations, allowing for marker-assisted selection and the generation of superior varieties. Consistent and overlapping associations provide confidence in the validity of the markers and associations. Further fine mapping and candidate gene analysis can be conducted to narrow down the genomic regions and identify specific genes associated with the trait. Secondary mapping populations have been utilized in crops such as rice, maize, wheat, tomato, cotton and soybean to map and confirm QTLs connected to characteristics such as disease resistance, yield potential, fruit quality features, and abiotic stress tolerance (Wissuwa et al., 2002; Niu et al., 2017; Chen et al., 2018).

The primary goal of NAM (Nested Association Mapping) is to combine the advantages of linkage mapping with association mapping. It involves creating a population of recombinant inbred lines (RILs) by crossing a common parent with multiple diverse founder parents. It plays a pivotal role in crop breeding by combining the benefits of linkage and association mapping, enabling trait dissection, facilitating marker-assisted selection, broadening the genetic base, and serving as a resource for functional genomics. These contributions enhance our understanding of complex traits, accelerate breeding efforts, and drive the development of improved crop varieties with desirable agronomic and quality traits. Researchers may locate sections of the genome related to certain qualities of interest by phenotyping the NAM population and genotyping it using high-density markers (Yu et al., 2008; Gage et al., 2020).

### 3.4 TILLING (targeting induced local lesions in genome)

TILLING is a non-transgenic high-throughput method (Henikoff et al., 2004). It involves the creation of chemically induced point mutations in the genome with PCR-based screening of the hetero duplex formed for crop improvement (McCallum et al., 2000a). The word “targeting” refers to focusing on genes of interest, usually taken as 1,500 bp in a single reaction. This technique overcomes the limitations associated with other tools of reverse genetics like insertional mutagenesis, RNA interference, and transposons (McCallum et al., 2000b). TILLING can also be used to study naturally present single nucleotide polymorphism in the genes of interest as a variant named Eco-TILLING (Gilchrist et al., 2006; Nieto et al., 2007; Ibarra et al., 2017). TILLING technique was first discovered by McCallum in the late 1990s who worked on Arabidopsis to characterize the two chromo methylase (*CMT 2*) gene functions. The goal of TILLING is to introduce specific changes into the DNA of crops to improve their traits, such as resistance to pests and diseases, increased yield, and improved nutritional content (Wang et al., 2012; Raihan et al., 2016a; Acevedo-Garcia et al., 2017) (Table 2). The process involves chemically inducing errors in the DNA and then screening for plants with the desired mutations. TILLING can be used in conjunction with CRISPR/Cas9 to deliver precise modifications to the genome and achieve particular breeding goals.

**TABLE 2:** Application of advanced breeding techniques in crop improvement.

Crops	Traits improved	Target genes	Remarks	References
QTL Mapping/Marker assisted selection				
Rice	Blast resistance	*Pi9, Pi2*	Blast resistance is imparted by the hybrid of Hui 316 (restorer line) and *Pi9, Pi2*, respectively	Tian et al. (2019)
Wheat	Drought tolerance	QTL found on chromosome 2A	QTLs for cell membrane stability, water content, and photosynthesis	Malik and Malik (2015)
Maize	Earliness and yield	QTL	Chromosomes 5, 8, and 10 have QTLs	Bouchez et al. (2002)
	European corn borer and Mediterranean corn borer	*42 SIR MQTL*	The two chromosomes with the highest SIR *MQTL* are 2 and 5. Fibre and hydroxycinnamate are cross-linked to prevent mechanical harm from insects	Badji et al. (2018)
	Maize rough dwarf disease (MRDD)	QTL *qMrdd*	*QMrdd8* from X178 is introduced into top germplasm using a conventional technique and MAS.	Xu et al. (2020)
Association Mapping				
Wheat	Karnal bunt resistance	13,098 SNPs	Population size of 339	Gupta et al. (2019b)
Brassica	Improving yield	74 significant QTNs detected	important loci associated with seed per silique and thousand-seed weight across the chromosomes of rapeseed by QTL and GWAS studies	Khan et al. (2019)
Rice	Abiotic stress	*NAC42* acts as a transcription factor that regulates the expression of the nitrate transporter gene	NUE-related agronomic traits	Tang et al. (2019)
Maize	Abiotic stress	Identified candidate genes associated with phosphorus deficiency tolerance	Metabolites under low Pi	Luo et al. (2019a)
TILLING Approach				
Wheat	Powdery mildew resistance	*TaMlo*	Partial loss-of-function	Acevedo-Garcia et al. (2017)
Wheat (*Triticum turgidum*)	Increased in amount of amylose	*SBEIIa*	The amalgamation of two non-sense mutations leads in a high amylose phenotype	Sestili et al. (2015)
Rice	Salt tolerance	*OsAKT1, OsHKT6, OsNSCC2, OsHAK11* and *OsSOS1*	Variation in membrane transport genes (expression levels and protein structures)	Hwang et al. (2016)
Barley	Changed starch phenotype	*MY1, GBSSI, LDA, SSI* and *SSIIa*	29 novel alleles were discovered in five genes linked to starch metabolism that are active in the endosperm during grain filling	Sparla et al. (2014)
Groundnut	Drought tolerance	*PLD*	Phospholipase D expression increase	Guo et al. (2015)
Accelerated Plant Breeding Approach (Speed breeding)				
Rice	Achieved 4–5 generations in a year (Salt tolerance)		Speed Breeding	Rana et al. (2019)
				Collard et al. (2017)
Wheat	Achieved 4–6 generations in a year			Mukade (1974)
				Watson et al. (2018)
Oat	Achieved 7 generations in a year			Liu et al. (2016)
Chickpea	Achieved 4–6 generations in a year			O’connor et al. (2013)
*Brassica napus*	Achieved 5 generations in a year			Watson et al. (2018)

## 4 Accelerated plant breeding techniques

**FIGURE 5:**
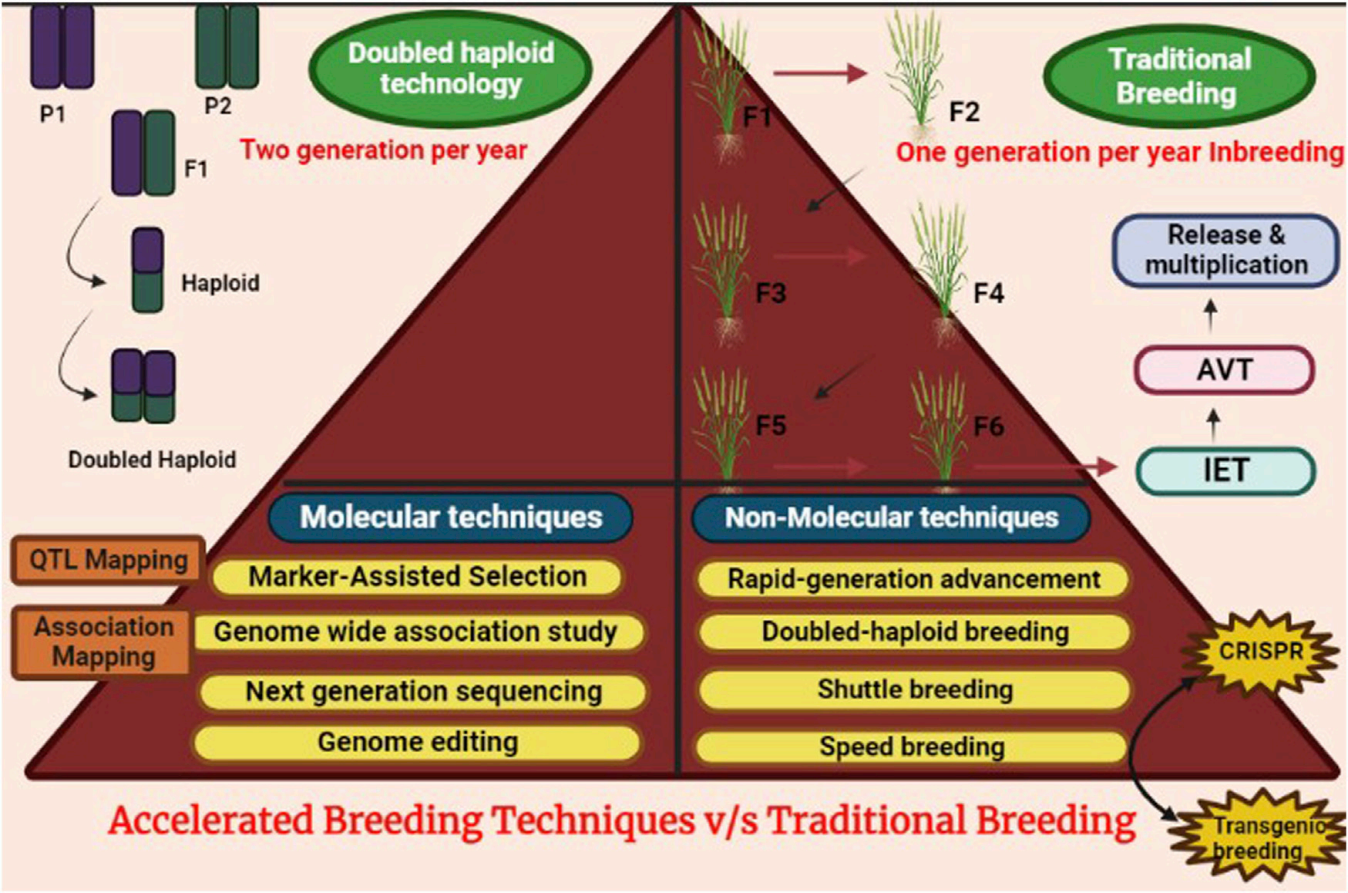
Comparison of accelerated breeding methods v/s traditional methods.

Traditional breeding is a time cumbersome process that includes different important phases. Three to 7 years are required for crossing and inbreeding to develop homozygous stable lines. Thereafter, four to 5 years of testing and selection for traits like quality, pest and disease resistance and yield are performed. Lastly, one to 3 years are required before release; in the multiplication of seeds. However, the period was reduced to half with the introduction of methods like shuttle breeding (Rajaram et al., 2002; Ortiz et al., 2007), which allowed the screening of 2 generations/year instead of one generation (Figure 5). Another technology that rapidly produced inbred lines without self-pollination cycles is doubled haploid technology (Ren et al., 2017; Wu et al., 2020). This method decreased the time for inbred making from 7 to 2 years. Nevertheless, there are several limitations to using this technology. For instance, there is no scope for early generation selection, lack of recombination and its effectiveness varies across crosses. While considering the larger scale of breeding programs, doubled haploid breeding turns out to be expensive (Chaudhary et al., 2019b; Chaikam and Prasanna, 2020).

Speed breeding innovation was stimulated by NASA, aiming to cultivate wheat in space. This technique utilizes specialized greenhouse facilities and carefully controlled lighting regimes to provide optimal growth conditions, allowing for the rapid acceleration of the breeding process. Usually, 22 h of light phase at around 22°C and 2 h of the dark period at 17°C are given to promote early flowering (Watson et al., 2018). For crops such as Triticum, Hordeum and Cicer spp., speed breeding can accomplish up to six cycles per year, resulting in faster selection and development of new cultivars with improved traits such as yield, disease resistance, and tolerance to environmental stress (Ghosh et al., 2018; Samineni et al., 2020). Although for crops like canola 4 generation/year can be achieved, which is still commendable, while working for pod-shattering phenotyping (Watson et al., 2018). Therefore, speed breeding has huge scope when it comes to doubling genetic gain and accelerated transfer of new alleles into adapted material through rapid backcrossing. Tremendous research is still going on in developing protocols for different crops like pepper, cassava, amaranthus, etc. (Stetter et al., 2016; Souza et al., 2018; Borovsky et al., 2020) (Table 2). DS Faraday was the first wheat variety produced via speed breeding that had excellent protein milling quality and resistance to PHS. It was released in partnership with DOW Agrosciences. The technique involves repeated cycles of selection for grain dormancy and backcrossing (Schwager, 2017). One of the key advancements in speed breeding’s future is its integration with CRISPR technology and the enhancement of transgenic techniques. Speed breeding will be closely linked with the revolutionary gene-editing tool CRISPR, allowing for more precise and efficient modifications in plant genomes (Murovec et al., 2018; Bao et al., 2020).

## 5 Phase III: genome editing technologies

This is a form of genetic manipulation in which DNA is incorporated to, or removed/substituted from a living organism’s genome for any desirable trait expression. In contrast to genetic engineering, which inserts the target gene into the host organism at random, genome editing targets the introduction to certain predefined locations. A gene knockout occurs when a frameshift mutation occurs in a gene, resulting in the cell no longer expressing any functional protein. ZFNs, TALENS and CRISPR can be used for gene knockouts. A gene knockdown is when a gene expression is reduced but not eliminated. This is usually accomplished by degrading or inhibiting the gene’s mRNA transcript from being translated.

### 5.1 Exploitation of natural DNA repair system in the host organism (through ZFNs, TALENs, and CRISPR)

Using appropriate genome editing technology techniques, double-stranded breaks may be generated. These DNA breaks stimulate the cellular DNA repair processes, allowing site-specific genomic changes to be introduced more easily (Rouet et al., 1994; Choulika et al., 1995). Artemis’ unique nuclease actions result in the formation of INDELs, rendering non-homologous end-joining repair systems inappropriate for precise alterations (Chang and Lieber, 2016). In situations where Homology-Directed Repair (HDR) is involved, a process that relies on a matching pair of chromosomes, the preservation of sequence information during the repair process is exceptionally high, exhibiting either minimal loss or no loss at all (known as the conservative type). At the site of the DSB, a required gene from the donor DNA strand is inserted using the homologous chromosome. Mammalian cells were formerly presumed to repair potentially lethal chromosomal double-strand breaks (DSBs) in part by non-homologous processes. Yet, it was later discovered that DSBs can increase homologous recombination by three or four orders of magnitude, suggesting that homology-directed repair is possible (Liang et al., 1998). As a consequence, the DNA-repair mechanism might be employed to insert the requisite genetic material, allowing for high-precision genome editing of a target cell. This type of genome editing can be employed to insert new genes or wipe out existing ones (Mali et al., 2013). Genome editing technologies can be categorized into four major groups: 1) Meganucleases; 2) ZFNs; 4) TALENs; 4) CRISPR/Cas9 system. However, the CRISPR/Cas9 system predominates as the preeminent and extensively employed methodology for genome editing.

The term “mega” is used to describe a massive recognition site. Because they are endonucleases, this location usually only appears once in each genome (Gallagher et al., 2014). The LAGLIDADG family contains the most well-known meganucleases proteins. LAGLIDADG proteins have one of two main functions 1) function as RNA maturase and 2) cleaving the exon-exon junction sequence where their intron is located, earning them the nickname “homing endonuclease.” This approach has a significant benefit in terms of safety since it is less harmful to cells than other naturally occurring restriction enzymes. Nevertheless, this procedure is both expensive and time-consuming.

Construction of the first chimeric restriction endonucleases gene by linking the finger domain to the non-specific cleavage domain Fok 1(*Flavobacterium okeanokoites*) (Kim et al., 1996). Zinc fingers use a mix of cysteine and histidine residues to coordinate zinc ions. Each domain’s-helix (also known as the “recognition helix”) may create sequence-specific interactions with DNA bases. There were two distinct realms, one DNA-binding domain (zinc finger motifs), which is made up of a chain of two-finger modules and recognizes three nucleotide sequences of DNA (one amino acid), and another DNA-cleaving domain, which is made up of Fok I nuclease domain. This method is described to have rapid disruption and integration into any genomic loci. Also, it can create gene knockouts in multiple cell lines. As compared to mega nucleases, this can have an off-target effect, and construction is complex as it needs two zinc finger motifs and a nuclease to create double-strand breaks.

Transcription Activator-Like Effector Nucleases (TALEN), like zinc finger nuclease is chimera that includes TALEs and Fok 1 endonuclease. The ability of TALENs to bind to DNA and promote the expression of their target genes by mimicking eukaryotic transcription factors is used as a DNA binding domain, in conjunction with the cleaving domain Fok 1, which causes double-stranded breaks. As compared to the Mega nucleases and zinc finger nuclease (ZFNs), Transcription Activator-Like Effector Nucleases TALEN is considered to have a simpler design and a higher specificity. However, two caveats were recognized in this technique 1) challenging to use in viral systems due to large protein size and 2) repetitive sequences may induce undesirable recombination events within the TALEN array.

The acronym CRISPR for Clustered Regularly Interspaced Short Palindromic Repeats was first time used in 2002 by Jansen et al. (2002). The word repeats refer to palindromic sequences which are interspaced by unique sequences called spacer. These spacers are molecular records in bacteria that are part of the virus genome which has earlier attacked the bacteria. This system works like an antigen/antibody system. The spacer is formed from protospacer sequences present in the virus which gets into the bacterial genome in the form of unique spacer sequences. Therefore, CRISPR is referred to as an adaptive immunity system. This system consists of RNA molecules and Cas enzymes that work together to identify and cut specific DNA sequences.

CRISPR-associated protein (Cas9) is an RNA-guided endonuclease that uses a single-guide RNA to cleave DNA at specific target sites. CRISPR/Cas9-based genome editing relies on creating a double-strand break in the DNA and then utilizing the cell’s natural DNA repair mechanisms. Within the native CRISPR/Cas9 system, the mature crRNA and transactivating crRNA come together to form a complex called tracrRNA: crRNA (Zhan et al., 2014). This complex serves as a guide for Cas9, directing it to the desired location on the DNA. While Cas9 is widely known, there are other forms of Cas proteins with distinct properties and functions. For example, Cas1 and Cas2 are involved in the adaptation phase of CRISPR systems, while Cas3 plays a role in the destruction of foreign DNA. Cas12 (Cpf1) is another variant that cleaves DNA with staggered ends, and Cas13 proteins target and cleave RNA molecules instead of DNA. Scientists have adapted this system for use in a wide range of organisms to precisely and efficiently edit their genomes. The ability to edit genes with unprecedented precision and ease has opened up many new possibilities for basic research and applied biotechnology.

CRISPR/Cas technology has significant potential for crop improvement by enabling precise and targeted genetic modifications in crops (Table 3). This technology has several advantages over traditional breeding methods, including speed, precision, and accuracy. With CRISPR/Cas, scientists can target specific genes in crop plants and make precise modifications, such as creating mutations or introducing new traits, without the need for introducing foreign DNA. This system consists of RNA molecules and Cas enzymes that work together to identify and cut specific DNA sequences. Scientists have adapted this system for use in a wide range of organisms, including plants, to precisely and efficiently edit their genomes. The ability to edit genes with unprecedented precision and ease has opened up many new possibilities for basic research and applied biotechnology. One application of CRISPR/Cas in crop improvement is to enhance the nutritional value of crops (Zhu et al., 2019). For example, researchers have used CRISPR/Cas to increase the iron content in rice, which is a significant dietary source of iron for many people. While CRISPR/Cas technology holds tremendous potential for genome editing, there are also some limitations and challenges that need to be addressed (Chen et al., 2021b; Yumlu and Stumm, 2021). This involves off-target effects (sometimes cut DNA at unintended locations, leading to unintended mutations), mosaicism, challenges in delivering methods, etc. Despite these challenges, researchers and companies around the world are working to overcome these limitations and leverage the potential of CRISPR/Cas technology for a wide range of applications.

**TABLE 3:** Application of genome editing in crop improvement.

Target gene	Trait improved	Remarks	References
Rice			
(*Hpt*) Hygromycin phosphotransferase		ZFNs	Cantos et al. (2014)
Resistance to bacterial blight		TALENs	Li et al. (2012)
Fragrant rice			Shan et al. (2015)
Resistance to disease & tolerant to abiotic stresses		CRISPR/CAS9	Xie and Yang (2013)
Increase resistance to blast			Liu et al. (2012)
Cold resistance resistant			Shen et al. (2017)
Tiller spreading			Miao et al. (2013)
Increase in grain number, grain size with thick erect panicles			Li et al. (2016a)
High amylose content			Sun et al. (2017)
Production of haploid plants			Yao et al. (2018)
Resistance to rice root-knot nematode		CRISPR/CAS9	Huang et al. (2023)
Reduce Cd accumulation			Chen et al. (2023)
Improving fragrance efficiency			Imran et al. (2023)
Agronomic traits and starch composition			Zheng et al. (2023)
Increase Photosynthesis			Caddell et al. (2023)
Broad-spectrum disease resistance			Liu et al. (2023)
Herbicide resistance		CRISPR/CAS9	Sun et al. (2016)
			Endo et al. (2016)
			Butt et al. (2017)
			Li et al. (2016b)
			Shimatani et al. (2017)
Nutritional quality improvement		CBEs	Li et al. (2017a)
Enhance nitrogen use efficiency			Lu and Zhu (2017)
Regulate senescence and death			Zong et al. (2017)
Resistance to blast			Ren et al. (2018)
Defence response			Ren et al. (2018)
Pathogen-responsive gene		ABEs	Yan et al. (2018)
Della protein for plant height			Hua et al. (2018)
Regulation of architecture of plant and grain yield			Hua et al. (2018)
Amylose synthesis			Hao et al. (2019)
Defence response			Hao et al. (2019)
Wheat			
*TaMLO*	Powdery mildew disease resistance		Shan et al. (2014)
*TaDREB2*	Dehydration responsive element		Shan et al. (2014)
*TaERF3*	Ethylene responsive factor		Shan et al. (2014)
*TaGW2*	Negative regulator of grain traits		Wang et al. (2018b)
*EDR1*	Resistance to powdery mildew		Zhang et al. (2017)
*TaSPL13*	Improve multiple agronomic traits		Gupta et al. (2023)
*SPO11-1*	Fertility and synapsis		Hyde et al. (2023)
*Tamyb10*	Pre-harvest sprouting-resistant red wheat		Zhu et al. (2023)
*Ppd-1*	Spike architecture		Errum et al. (2023)
Lipid metabolism		CBEs	Zong et al. (2017)
Panicle length and grain weight		ABEs	Li et al. (2018b)
Maize			
*ZmIPK1*	Responsible for herbicide tolerance and reduction of phytate content	ZFNs	Shukla et al. (2009)
*ZmGL2*	Reduced epicuticular wax in leaves		Char et al. (2015)
*ZmMTL*	Production of haploids	TALENs	Kelliher et al. (2017)
*ARGOS8*	Expressed well under drought stress with increase in grain yield		Shi et al. (2017)
*ZmIPK1A, ZmIPK* and *ZmMRP4*	Phytic acid synthesis		Liang et al. (2014)
*PSY1*	Phytoene synthase		Zhu et al. (2016)
*Zmzb7*	Knockout of gene resulted in albino plant		Feng et al. (2016)
*ZmTMS5*	Thermosensitive genic male-sterile		Li et al. (2017b)
*Wx1*	High amylopectin content		Pioneer (2016)
*ALS*	Herbicide resistance		Svitashev et al. (2015)
*ARGOS8*	Drought stress tolerance		Shi et al. (2017)
*ipdC*	Promote maize growth		Figueredo et al. (2023)
36 genes potentially involved in leaf growth	10% increase in leaf size		Impens et al. (2023)
*pipeline BREEDIT*	Improve complex traits such as yield and drought tolerance		Lorenzo et al. (2023)
First time multiplex gene editing		CRISPR/Cas9 (tRNA-RNAprocessing system)	Qi et al. (2016)
Chromosomal segregation		CBEs	Zong et al. (2017)
Tomato			
Production of purple tomatoes		TALENs	Cermak et al. (2015)
Powdery mildew resistance		CRISPR/Cas9	Nekrasov et al. (2017)
Bacterial speck resistance			Ortigosa et al. (2019)
Tomato domestication			Li et al. (2018c)
Earlier harvest time			Soyk et al. (2017)
Parthenocarpy			Klap et al. (2017)
Repression of fruit ripening			Ito et al. (2015)
Prevents tomato fruit ripening			Yang et al. (2017b)
Increase shelf life			Yu et al. (2017)
Leaf shape variations and seedless fruits			Ueta et al. (2017)
Drought tolerance			Wang et al. (2017)
*PSY1, MYB12,* and *SGR1*	Fruit colour-related genes		Yang et al. (2023)
*SlATG5*	Resistance to Botrytis cinerea		Li et al. (2023)
*SlHyPRP1*	Multi-stress tolerance		Tran et al. (2023)
*SlDYT1* and *SlGSTAA*	Male Sterility		Zhou et al. (2023)
Herbicide resistance		CBEs	Veillet et al. (2019)
*SlRIN*	Tomato fruit ripening	ABEs	Niu et al. (2023)
Soyabean			
High oleic acid contents		TALENs	Haun et al. (2014)
High oleic & low linoleic contents			Demorest et al. (2016)
Herbicide resistance		CRISPR/Cas9	Li et al. (2015)
Disease resistance against *Phytophthora sojae*			Fang and Tyler (2016)
Flowering time			Cai et al. (2018)
Carotenoid biosynthesis			Du et al. (2016)
Potato			
Minimizing reducing sugars		TALENs	Clasen et al. (2016)
Herbicide resistance		CRISPR/Cas9	Butler et al. (2016)
High amylopectin content			Andersson et al. (2017)
Herbicide resistance			Veillet et al. (2019)
*VInv*	Quality of potato tubers		Sattar et al. (2023)
*VInv* and *AS1*	Reduced Browning		Ly et al. (2023)
Sugarcane			
Improved cell wall composition		TALENs	Jung and Altpeter (2016)
Improved efficiency of saccharification			Kannan et al. (2018)
Arabidopsis			
*MIR169a*	Drought tolerance	CRISPR/Cas9	Zhao et al. (2016)
Turnip mosaic virus (TuMV) resistance		CRISPR/Cas9	Pyott et al. (2016)
Increased stomatal closure against abscisic acid		CRISPR/Cas9	Osakabe et al. (2016)
High-light acclimation and photomorphogenesis		CRISPR/Cas9	Atanasov et al. (2023)

### 5.2 Base editing

After the discovery of CRISPR/Cas9 system, another mechanism called base editing has also become a popular tool for genome editing. Base editing is revolutionizing crop improvement by allowing alteration (Gaudelli et al., 2017) of the genome by making specific changes to the DNA base pairs (Komor et al., 2016; Porto et al., 2020). This technology can be used to change one DNA letter into another, which can then lead to new traits or characteristics appearing in the plants (Henikoff and Comai, 2003). Base editors can make precise, single-nucleotide changes to the genome without inducing double-stranded breaks (Rees and Liu, 2018; Molla and Yang, 2019). This makes base editing an attractive alternative to traditional CRISPR/Cas9 methods, which often result in unwanted indels. Base editors are made up of two components: a CRISPR protein that can be configured to target a particular position in the genome and an enzyme that can chemically alter the DNA base at that position. The most often used base editors are cytosine base editors (CBEs) and adenine base editors (ABEs), which are used to change a C-G base pair to a T-A base pair or an A-T base pair to a G-C base pair, respectively. CBEs use a modified version of the CRISPR protein Cas9 that has been fused to a cytidine deaminase enzyme (Li et al., 2018a). The deaminase enzyme converts the C base to a U base, and then the DNA repair machinery in the cell converts the U to a T, resulting in a C to T base change (Zhang et al., 2020). ABEs, on the other hand, employ a separate enzyme known as an adenine deaminase to convert an A base to an inosine base, which the DNA repair machinery interprets as a G base, resulting in an A to G base conversion (Kang et al., 2018; Hua et al., 2020).

The benefits of base editing include its precision, efficiency and lack of off-target effects (Lee et al., 2020). Base editing has already had a positive impact on crop production, and it is predicted to play a more significant role in the future as more genes are discovered that can be edited with this technique (Eid et al., 2018). Crop improvement by base editing is a technique that entails amending DNA to enhance its attributes (Bharat et al., 2020). This can be done by either mutating existing genes or adding new ones. Base editing is different from traditional methods of genetic modification, as it does not require the use of foreign DNA. This overcomes regulatory issues (Jones, 2015). There are several ways in which base editing can be used to improve crops. One way is by increasing resistance to biotic and abiotic stress (Zhang et al., 2018a; Sood et al., 2022). This can be done by altering the genes that encode proteins that are targets of disease-causing organisms. Another way is by improving the nutritional value of crops. This can be done by modifying the genes that control the production of vitamins and minerals (Kumar et al., 2021).

### 5.3 Prime editing: unlocking the precision of genetic rewriting

It is a breakthrough advanced technique over existing CRISPR/Cas9 tools. Prime editing is complementary to base editing to correct small mutations including indels (Anzalone et al., 2019; Kantor et al., 2020). CRISPR/CAS9 as stated above causes double-stranded breaks. However, it was seen in a few studies that double-stranded breaks are not safe (Carusillo and Mussolino, 2020; Ochoa-Sanchez et al., 2021). It can cause mutagenic activity, a complex mix of undesirable products or may cause translocation of DNA. These breaks can trigger P53 activity which can induce cell death (Shen and Li, 2022). On the other hand, base editing is limited to four possible transition mutations (C to T, A to G, T to C, and G to A) and is still prone to off-target effects. Base editing has not proved to be useful in the case of mutations like insertions and deletions. Therefore, to overcome these obstacles, prime editing was developed (Marzec and Hensel, 2020).

Prime editors have three major components referred as pegRNA, CAS9 H840A nickase fused with M-MLV reverse transcriptase and single guide RNA. Guide RNA is lengthened including mutant target recognition sequences and correction sequences which in combination are referred as pegRNA. The CAS enzyme domains are also modified like in base editing such that only one strand is cut. Finally, instead of a base editing domain, a reverse transcriptase activity domain uses the corrected sequence of the peg RNA as a template to synthesize the corresponding stretch of the DNA strand. The newly synthesized stretch of DNA will then bind to the untampered original DNA along with creating a flap that the stretch of new DNA is supposed to replace. The flap will be cut out since it is an unnatural DNA structure. Although a new segment of DNA contains the correct DNA sequence, the other segment still does not. This mismatch will be corrected by a triggered natural repair mechanism (Marzec et al., 2020; Molla et al., 2021; Hillary and Ceasar, 2022). Its major inference lies in the precise gene editing with reduced off-target mutations and wider scope of applications. This technique can bring revolution by allowing for precise alterations to specific genes which will eventually increase yield by providing tolerance to biotic and abiotic stress. (Gao, 2021; Lu et al., 2022).

### 5.4 Achievements of genome editing in plant breeding

Genome editing in plant breeding has achieved several desirable outcomes (Table 2). Most significant is breeding for resistance in staple crops like rice and wheat by modifying the genes responsible for susceptibility to various diseases (Wang et al., 2014; Zhou et al., 2015; Wang et al., 2018b). Additionally, genome editing can modify genes that control yield-related traits (Ainley et al., 2013; Zhang et al., 2018a; Zong et al., 2018). For instance, researchers have altered a gene in rice that controls the plant’s sensitivity to nitrogen using CRISPR/Cas9, resulting in plants that yield more with less fertilizer. The nutritional content of crops may be improved through genome editing (Connorton et al., 2017). For instance, by altering the genes that produce beta-carotene in rice, scientists were able to produce a type of grain known as “golden rice” that had greater quantities of vitamin A (Busch and Schneeberger, 2019). Another important application is for adaptation to environmental stress. This may lead to crops that are more resilient to the difficulties posed by a changing climate (Zhang et al., 2014).

### 5.5 Unlocking nature’s blueprint: target genes for precision genome editing

CRISPR techniques have revolutionized genome editing and have made it possible to target any gene of interest with a high degree of precision. Researchers can modify genes involved in various traits, such as disease resistance, quality attributes, yield, and many other desired characteristics. For instance, *TaDREB2* is a transcription factor that plays a crucial role in drought stress response in wheat. It is involved in regulating the expression of stress-responsive genes that help the plant cope with water deficit conditions. Additionally, *TaERF3* is another transcription factor that is associated with abiotic stress responses, including drought, heat and salinity stresses in wheat (Kim et al., 2018). In maize, the *mLG1*, *UB2* and *UB3* genes have been utilized for the development of a haploid-inducer mediated genome editing system (Wang et al., 2019a). The *mLG1* gene is a maternally expressed gene that plays a role in inhibiting embryo development when it is paternally inherited. The *UB2* gene is a ubiquitin-conjugating enzyme that is involved in protein degradation and regulation. The *UB3* gene is a ubiquitin ligase that plays a role in the degradation of proteins through the ubiquitin-proteasome system. In tomato crops, one of the target genes for improving quality traits is *ACS2* (Ito et al., 2021)*.* The purpose of editing this gene is to regulate the ripening process and extend the shelf life of tomatoes (Peng et al., 2022). For herbicide tolerance, the target gene is *EPSPS* (5-enolpyruvylshikimate-3-phosphate synthase). By editing this gene, scientists aim to confer resistance to the widely used herbicide glyphosate (Achary et al., 2020; Wang et al., 2021a; Li et al., 2021). CRISPR technology provides a powerful tool for gene editing (Table 3), but there are still challenges to overcome, such as off-target effects and ensuring precise and accurate edits. However, ongoing research and advancements in CRISPR techniques continue to improve the precision and efficiency of gene editing, making it a promising technology for various applications.

## 6 Phase IV: beyond and next-generation smart crops

These are sophisticated agricultural technologies and approaches that increase crop productivity, efficiency, and sustainability. This includes various techniques like Haplotype crop breeding, genomic selection, use of secondary population, artificial intelligence (AI), etc.

### 6.1 Genomic selection

Genomic selection utilizes genomic information to predict the performance of plants and select the best individuals for breeding. Genomic selection has been used in a variety of crops to advance breeding programmes and increase crop improvement efficiency (Benavente and Giménez, 2021). Genomic selection, for example, has been used in cereal breeding to improve yield, drought tolerance, disease resistance, and nutritional quality (Haile et al., 2020; Simmons et al., 2021). Breeders can find maize lines with high genomic estimated breeding values (GEBVs) for these characteristics by utilizing genomic information and marker-assisted selection, accelerating the production of better varieties (Crossa et al., 2017). GS method facilitates the early selection of individuals with favorable genomic profiles, resulting in the production of high-performing wheat cultivars (Sinha et al., 2021). By harnessing the power of genomics, breeders can make more accurate selections, enhance breeding efficiency, and develop improved crop varieties with desired traits.

### 6.2 Haplotype-based breeding

It is a cutting-edge technology that entails discovering and choosing precise allele combinations within certain genomic areas known as haplotypes to obtain desired crop attributes (Bhat et al., 2021). For instance, in wheat the method may be used to boost disease resistance against rusts and Fusarium head blight, as well as attributes such as drought tolerance and yield potential (Athiyannan et al., 2022; Alemu et al., 2023). Advances in genomic technologies, such as high-throughput genotyping and genome sequencing, have facilitated the identification and characterization of haplotypes in rice. This has opened up new opportunities for breeders to accelerate the development of improved rice varieties by incorporating favorable haplotypes into their breeding programs. It helps in developing blast-resistant, bacterial blight-resistant, and submergence-tolerant cultivars while also increasing grain quality traits (Tanweer et al., 2015; Verma et al., 2023). Few studies have highlighted the use of haplotype analysis to understand germplasm diversity in maize breeding programs. Haplotype-based approaches provide advantages over single-marker-based methods in assessing population structure and capturing additional information compared to individual SNPs (Coffman et al., 2020). Improved features in maize include drought tolerance, insect resistance, and nitrogen usage efficiency (Simmons et al., 2021). Similarly, haplotype-based breeding may be used to improve disease resistance, qualitative traits, and stress tolerance in diverse crops such as tomato, potato, cotton, and barley. Various studies have discussed the advantages of haplotype-based breeding, such as its ability to capture the combined effects of multiple genetic variants and its potential to increase the accuracy of trait selection.

### 6.3 Omics-based breeding

Omics-based plant breeding is the use of high-throughput technology and data-driven methodologies for investigating and altering plant genetic and molecular features for crop development. Omics-based approaches have found successful applications in various crops, and one notable example is in the field of rice breeding (Dai et al., 2022; Zaghum et al., 2022). Genomics has proven critical in decoding the rice genome sequence and discovering genes linked to crucial agronomic features (Peng et al., 2020). Transcriptomic studies on rice have shown gene expression patterns at various developmental stages and stress responses. Understanding the activities of individual proteins involved in grain quality and stress tolerance processes has been improved by proteomics (Choi, 2019). Metabolomics has aided in the discovery of compounds associated with nutritional characteristics and stress responses in rice. Overall, in crop breeding programmes, these omics-based techniques have aided in the identification of candidate genes, molecular markers, and important pathways linked with desirable features (Alotaibi et al., 2021; Cao et al., 2022; Shen et al., 2022).

## 7 Convergence of genome-assisted breeding and genome editing

The confluence of genome-assisted breeding and genome editing entails merging genomic information with precise gene editing methods to boost agricultural yield. Genome-assisted breeding assists in finding significant genes linked with desired qualities, while genome editing enables precise gene alteration. By combining these techniques, breeders can minimize the time and resources necessary for the production of superior cultivars with increased attributes, as well as enable the transfer of beneficial genetic variants from wild relatives. This confluence provides a tremendous tool for expediting crop development efforts and tackling agricultural concerns.

## 8 Empowering food security with resilient orphan crops through diverse breeding approaches

A range of orphan crop species exhibit regional significance and possess stress resilient traits in order to thrive extreme climatic conditions owing to their relevant genes and stress combating mechanisms. However, these species lack global trade and substantial recognition from a research standpoint. Hence, research initiatives attempt to exploit their potential to improve major crops, address nutritional challenges and enhance food system sustainability (Dawson et al., 2019; Yaqoob et al., 2023). Lemmon et al. (2018) elucidated the viability of different breeding approaches and underscored the significance of allocating resources to study and acquire further knowledge concerning genomes, genes, and cellular mechanisms underlying plant characteristics. Several conventional as well as advanced breeding techniques have been employed to enhance the desirable traits of orphan crops yet.

Historically, the genetic enhancement of orphan crops were primarily restricted to conventional breeding practices, employing pedigree-based selection methods to enhance desired traits such as larger seed size, increased yield, ease of propagation, reduced seed dispersal, etc. The emphasis was predominantly placed on improving the domestication process of these crops (Kamenya, et al., 2021). For orphan legumes (pea, lupin) mutation breeding and interspecific introgression have been successful in generating genetic diversity leading to favorable traits like stress tolerance, high yield, etc., (Chongtham et al., 2022). A robust repertoire of molecular markers serves as a crucial asset in the breeding endeavors of all crop species; however, it is often deficient in broad array of orphan crops. Among the techniques employed for molecular marker development in orphan crops, Diversity Arrays Technology has emerged as a highly significant method. The advent of this technology ushered in a paradigm shift in the genetic profiling and establishment of genetic linkages in several crops (predominantly pigeon pea and cassava) that were previously deemed as orphaned, a span of approximately 20 years ago (Kamenya, et al., 2021). However, with the sequencing of more than 35 orphan crops, rapid SNP discovery has become possible (finger millet, Bambara groundnut, lupin, etc.). Certain researchers have pinpointed specific genes or quantitative trait loci (QTLs) associated with adaptive traits in orphan legume species. These genetic elements possess the potential to be utilized in crop enhancement efforts to confer stress tolerance onto other cultivated crops (Chongtham et al., 2022). Although research on speed breeding in orphan crops is still limited, there are a few examples where it has been explored. For instance, chickpea, peanut and amaranth speed breeding protocol has been developed (Chiurugwi et al., 2019). Speed breeding could be used to shorten the breeding cycle of fonio and facilitate the development of improved varieties with increased yield, disease resistance, and drought tolerance (Ibrahim Bio Yerima and Achigan-Dako, 2021). Some initiatives have been there for implementing TILLING in various orphan crops like pearl millet, teff, cassava, mung bean, chickpea, banana, etc. (Esfeld et al., 2013). Genome editing has been exploited in orphan crops like sorghum (modulating flowering time), foxtail millet (male sterility), chickpea (draught tolerance), etc. (Venezia and Creasey Krainer, 2021).

These days, with the advent of genome editing techniques, these underutilized crops are undergone targeted improvements, leading to advancements in their characteristics and overall performance (Table 4). Enhancing essential nutrients such as iron, zinc, a range of vitamins can have significant impacts on improving the nutritional quality of diets that heavily rely on these crops (Tadele, 2018). A study showed that in the future, the CRISPR/Cas9 tool holds potential for application in the Ca transporter genes of finger millet. The docking study proposed that *EcCBL4* has a strong binding affinity with *EcCIPK24* and might play a significant role in the accumulation of Ca in seeds (Chinchole et al., 2017). Classical domesticated genes possess characteristics that make them ideal candidates for Cas base editing. They are well characterized, exhibit simple genetic architecture, and typically have a monogenetic nature -(Rasheed et al., 2021). Studies have shown the role of TALENs to target a gene involved in the lignin biosynthesis pathway in pearl millet. By knocking out this gene, they achieved improved resistance against downy mildew, a significant foliar disease in pearl millet (Maurya et al., 2022). Cassava yield is significantly affected by disease-causing pathogens nearly around 50% of total yield loss attributed to the African cassava mosaic virus (ACMV) and cassava brown streak disease (CBSD). Hence, targeted mutation using Cas9/gRNA, have been developed to address such challenges. Orphan crop genomes were annotated through whole-genome sequencing and their transcriptomes were generated. In the annotation process, transcriptomes from the same species were preferred, as seen in examples like the african eggplant, wild mustard, tef, etc. (Cannarozzi et al., 2014; Bhardwaj et al., 2015; Chen et al., 2021c). However, in some cases, closely associated or model crop transcriptomes were used, as in finger millet, which utilized data from maize. RNA sequencing (RNA-seq) became the method of choice for generating specific crop transcriptomes (Ozsolak and Milos, 2011). Microarrays were the preferred method before NGS technology for transcriptome analysis in various orphan crops to uncover expression profiles associated with abiotic stress resilience. Among the crops studied were buckwheat, tef (Golisz et al., 2008), white lupine (Zhu et al., 2010), etc. Another study detected 2,416 DEGs in quinoa (*Chenopodium quinoa*) during salt stress profiling (Ranasinghe et al., 2019). Additionally, transcription analysis in jute-mallow helped identify genes related to drought stress response (Yang et al., 2017a).

**TABLE 4:** Potential role of genome editing in orphan crops.

Crop	Target gene	Trait improved	Remarks	References
Sorghum	Alpha-Kafirin gene family	Increase digestibility and protein quality	CRISPR/Cas	Li et al. (2018d)
	FLOWERING TIME (*FT*); Gibberellin 2-oxidase 5 (*Ga2ox5*)	Flowering time		Char et al. (2020)
Finger millet	*Bhlh57*	Salinity resistance		Babitha et al. (2015)
Foxtail millet	Phytochrome C (*PHYC*)	Photoperiodic flowering		Yang et al. (2020)
	DROOPY LEAF1 (*DPY1*)	Plant architecture		Zhao et al. (2020)
	*SiMTL*	Haploid embryo induction		Cheng et al. (2021)
Sweet potato	Granule-bound starch synthase I (*GBSSI*)	Availability of more digestible sugars		Wang et al. (2019b)
	Starch branching enzyme II (*SBEII*)			
Cassava	Protein targeting to starch 1 (*PTST1*)	Increase in digestible sugars		Bull et al. (2018)
	*EPSPS*	Glyphosate tolerance		Hummel et al. (2018)
Pigeon Pea	*CcFT8*	Florigen producing gene		Tribhuvan et al. (2020)
Quinoa	*WUSCHEL, BABY BOOM* and *LEAFY COTYLEDON1*	Improve the transformation efficiency		Wang et al. (2021b)
Lettuce	GDP-L-galactose phosphorylase 1 (*GGP1*)	Increase in the vitamin C		Zhang et al. (2018b)
	GDP-L-galactose phosphorylase 2 (*GGP2*)			
Yam	Phytoene Desaturase (*PDS*)	Carotenoid biosynthesis		Syombua et al. (2020)
	*ERF* (ethylene-responsive factor)	Resistance to anthracnose		Ntui et al. (2021)
Eggplant	Polyphenol oxidase (*PPO*)	Decreased browning		Maioli et al. (2020)
Bambara groundnut	*KUP*	Abiotic stress tolerance		Sharma et al. (2022)
Fonio	*DeSh1-9A*	Reduced seed shattering		Abrouk et al. (2020)
Teff	*SEMIDWARF-1* (*SD-1*)	Semi-dwarfism and lodging resistance		Beyene et al. (2022)
	*OsSPL14*, *OsmiR397*	Panicle branching trait		Numan et al. (2021)
Watermelon	Acetolactate synthase (*ALS*)	Herbicide-resistant		Tian et al. (2018)

## 9 Conclusion and future thrust

The trend in plant breeding has evolved significantly over the years, shifting from conventional breeding to more advanced molecular breeding, and this shift is likely to continue in the future with novel biotechnology tools. Traditional breeding relies on the genetic diversity of the parent plants and necessitates maturing plants and waiting for the next-generation to be produced. While breakthroughs in biotechnology have substantially improved breeding precision and speed, a shortage of facilities and financial resources keeps conventional breeding still in demand. Many breeders now use a combination of conventional and molecular breeding techniques to produce crops with improved traits. This has led to the development of new crop varieties that are more resilient to disease, pests, and environmental stresses, with better yields and improved nutritional quality. In summary, the future thrust of plant breeding will involve developing crop varieties that are resilient to climate change, provide nutritional security, are sustainable, use precision agriculture techniques, utilize gene editing technologies, leverage genomics and big data, and involve collaborative research efforts.
